# Molecular genetic diagnosis and surgical management in a cohort of children with 46,XY disorders/differences of sex development

**DOI:** 10.3389/fped.2025.1456227

**Published:** 2025-01-28

**Authors:** Yuenshan Sammi Wong, Ho Ming Luk, Ho Chung Yau, Lap Ming Wong, Sarah Wing Yiu Poon, Joanna Yuet Ling Tung, Yuk Him Tam

**Affiliations:** ^1^ Division of Paediatric Surgery & Paediatric Urology, Department of Surgery, Prince of Wales Hospital, The Chinese University of Hong Kong, Hong Kong, Hong Kong SAR, China; ^2^ Department of Surgery, Hong Kong Children’s Hospital, Hong Kong, Hong Kong SAR, China; ^3^ Department of Clinical Genetics, Hong Kong Children’s Hospital, Hong Kong, Hong Kong SAR, China; ^4^ Department of Paediatrics, Prince of Wales Hospital, Hong Kong, Hong Kong SAR, China; ^5^ Department of Paediatrics & Adolescent Medicine, Tuen Mun Hospital, Hong Kong, Hong Kong SAR, China; ^6^ Department of Paediatrics & Adolescent Medicine, Hong Kong Children’s Hospital, Hong Kong, Hong Kong SAR, China

**Keywords:** disorders/differences of sex development, 46,XY, genetic diagnosis, surgery, gonadal dysgenesis

## Abstract

**Objective:**

A firm diagnosis revealing the etiology of disorders/differences of sex development (DSD) is most helpful in guiding clinical management. The aim of this study is to investigate molecular genetic diagnoses and surgical treatment in a cohort of children with 46,XY DSD.

**Methods:**

A retrospective study was conducted on children with 46,XY DSD. They were referred to a tertiary surgical center during the period between 2011 and 2022 and were found to have genetic alterations, which were considered etiologies for their DSD. Data on clinical presentations, sex of rearing, genetic findings, surgical treatment, and comorbidities were collected and reviewed.

**Results:**

A total of 21 patients were included in the study: 11 and 10 were reared as male and female, respectively. Genetic alterations were found as the causes for androgen insensitivity syndrome (*n* = 4), 5-alpha reductase type II deficiency (*n* = 5), 17-beta hydroxysteroid dehydrogenase III deficiency (*n* = 1), 17-alpha hydroxylase deficiency (*n* = 1), and gonadal dysgenesis (*n* = 10). Of those with gonadal dysgenesis, the genetic alterations were *NR5A1* mutation/deletion (*n* = 3), *DMRT1* deletion (*n* = 4), *WT1* mutation (*n* = 2), and *DAX1* duplication (*n* = 1). A total of 20/21 patients underwent one or more surgical procedures including hypospadias repair (*n* = 10), gonadectomy (*n* = 11), gonadal biopsy (*n* = 4), hernia repair (*n* = 4), orchidopexy (*n* = 1), and feminizing genitoplasty (*n* = 1). A total of 5/21 had germ cell neoplasms in one or both gonads. A total of 8/10 patients with gonadal dysgenesis had comorbidities involving other systems. Of the whole group, seven patients were found to inherit genetic alterations from their parents.

**Conclusions:**

Molecular genetic diagnosis enhances the understanding of etiology, improves diagnostic accuracy, and provides precise guidance in the counseling and surgical management of children with 46,XY DSD.

## Introduction

Sex development is a complex process that starts with the initial establishment of sex chromosomes followed by sex determination, in which bipotential gonads develop into either testes or ovaries. The final step is sex differentiation, where internal and external genitalia are formed, resulting in a phenotype typical for either male or female (1). Disorders/differences of sex development (DSD) are congenital conditions featuring atypia of chromosomal, gonadal, and phenotypic sex (2).

The Chicago Consensus classified DSD according to the karyotype to allow a broad diagnostic category to be reached early (2). Nonetheless, children with 46,XY DSD present with a wide range of phenotypes, from hypospadias and severe atypical external genitalia to female-typical external genitalia in the presence of a male karyotype. Ideally, a causative diagnosis should be reached in every case to individualize management. However, identifying the etiology in 46,XY DSD is challenging, and the cause in many cases remains unknown (3–5).

In recent years, the advantages of establishing a molecular genetic diagnosis for children with DSD have been increasingly emphasized (1, 6–8). Given the overlapping clinical phenotypes and biochemical features in 46,XY DSD, a genetic diagnosis is helpful in affirming the underlying cause and providing precise guidance in management. A genetic diagnosis may inform genotype–phenotype correlation and address the issues of recurrence risk for individuals and their families. Moreover, a genetic diagnosis may provide prognostic information regarding gonadal function, tumor risk, fertility potential, and gender identity (1, 7, 8).

Our diagnostic approach for DSD has been evolving with an increasing application of molecular testing in recent years. The purpose of the present study is to investigate the genetic etiology and surgical management of a cohort of children with 46,XY DSD who had been referred to a tertiary surgical center that was part of a multidisciplinary team (MDT) for DSD.

## Methods

### Patients and study design

This study was a retrospective review of medical records with the approval of our institutional clinical research ethics committee. We identified patients who had clinical presentations within the spectrum of DSD and were referred to our surgical center during the period between 2011 and 2022. The spectrum of DSD phenotypes ranged from micropenis, hypospadias with additional undervirilization features such as micropenis/undescended testes/bifid scrotum, severe atypical external genitalia, to female-typical external genitalia with palpable gonads or with discordant Y-chromosome. In this study, we included only those who had a karyotype of 46,XY, and molecular tests revealing relevant genetic alterations, which were considered etiologies for DSD. Data collected included the sex of rearing, primary presentations, clinical phenotypes, molecular test results, histological findings, surgical treatment, and comorbidities.

### Molecular genetic tests

There was no standardized protocol for genetic testing during the study period. Testing methods were based on clinical phenotypes, biochemical findings, and availability of testing modalities in clinical laboratories. All patients had the standard karyotype. In addition, patients during the early study period underwent single-gene testing by Sanger sequencing based on clinical and biochemical clues. With next-generation sequencing (NGS) becoming available in clinical settings from 2016, targeted DSD-related gene panels or whole-exome sequencing (WES) were used in selected cases. Chromosomal microarray or multiplex ligation-dependent probe amplification (MLPA) testing was performed to detect copy number variants if clinically indicated. The pathogenicity of genetic alterations was categorized according to the guidelines of the American College of Medical Genetics and Genomics (ACMG) (9). Genetic alterations that were identified as pathogenic variants, likely pathogenic variants, and variants of uncertain significance (VUS), which were strongly associated with the clinical, biochemical, and histological findings of patients, were reported as genetic diagnoses in this study.

### Study subjects categorization

The study subjects were categorized in accordance with the pathophysiological mechanism of 46,XY DSD and fell into one of three categories (2): (1) gonadal dysgenesis (GD), either partial (PGD) or complete (CGD), to describe disorders of gonad development; (2) androgen insensitivity syndrome (AIS), either partial (PAIS) or complete (CAIS), to describe disorders of androgen action; and (3) specific enzyme deficiency to describe disorders of androgen synthesis.

## Results

In total, 21 patients were identified and included in the study. All had the karyotype 46,XY. Of these, 11 and 10 were reared as male and female, respectively. Among those who presented at birth, the sex of rearing was determined through a shared-decision process between the MDT and the parents. Factors taken into consideration included definitive diagnosis, prediction of future gender development, anatomical features of external genitalia, fertility potential, and the need for subsequent medical and surgical treatment. Fourteen patients presented at birth with atypical genitalia, prompting investigations. Two presented with female-typical phenotype at birth with a discordant prenatal karyotype of 46,XY. The remaining five patients, all with female-typical phenotypes, presented with incidental findings of 46,XY karyotype during genetic workup for other comorbidities (*n* = 2), finding of testis during hernia repair (*n* = 1), primary amenorrhea (*n* = 1), and virilization at onset of puberty (*n* = 1).

Of the whole group, a molecular genetic diagnosis was reached in patients with disorders of androgen action (*n* = 4), disorders of androgen synthesis (*n* = 7), and disorders of gonad development (*n* = 10). All but one patient had pathogenic or likely pathogenic variants in DSD-related genes. The only VUS reported in this study occurred in a patient with GD. Clinical and genetic data of 21 patients are summarized in Table 1.

**Table 1:** Summary of the clinical and genetic data of 21 patients.

Patient no.	SOR	Phenotype (age at presentation)	Gene (age at genetic diagnosis)	Variant^a^ Amino acid change or chromosomal rearrangement, Zygosity, Testing techniques	Relevant biochemistry at birth or minipuberty or at the time of presentation	Clinical diagnosis	Surgery (age at time of surgery)	Co-M	Implications of genetic diagnosis
1	M	Proximal hypospadias, micropenis (at birth)	*SRD5A2* (7 months)	c.680G>A p.(Arg227Gln) Homo WES	Normal LH, FSH, and T USP result inconclusive	5α-R2D	TIP repair(20 months)		Genetic counseling Parental testing for carrier status and recurrence risk DHT cream before hypospadias surgery Prognostic prediction in favor of male gender Risk of infertility
2	M	Proximal hypospadias, micropenis (at birth)	*SRD5A2* (2 months)	c.364_365delAA p.(Asn122Trpfs*13) c.607G>A p.(Gly203Ser) Comp hetero Sanger	Normal LH, FSH, and T	5α-R2D	Staged repair of hypospadias (1-2 years)		
3	M	Proximal hypospadias, micropenis (at birth)	*SRD5A2* (3 months)	c.607G>A p.(Gly203Ser) c.680G>A p.(Arg227Gln) Comp hetero Sanger	USP suggestive of 5α-R2D	5α-R2D	TIP repair (2 years)		
4	M	Proximal hypospadias, micropenis, UDT (At birth)	*SRD5A2* (7 months)	c.680G>A p.(Arg227Gln) Homo Sanger	hCG stimulation Pre T/DHT = 7 Post T/DHT = 30.7	5α-R2D	TIP repair (17 months) orchidopexy (6 years)		
5	M	Proximal hypospadias, micropenis (at birth)	*SRD5A2* (2 years)	c.143C>T p.(Pro48Leu) c.737G>A p.(Arg246Gln) Comp hetero Sanger	Normal LH, FSH, and T	5α-R2D	Staged repair of hypospadias Lap hernia repair (3–4 years)		
6	F	Female phenotype, Bil palpable gonads in labia (at birth)	*HSD17B3* (2 months)	c.210del p.(Lys70Asnfs*16) Homo WES	LH↑T↓ for the male newborn AMH normal for the male infant	17βHSD-3 deficiency			Genetic counseling Parental testing for carrier status and recurrence risk Risk of virilization at puberty Prepubertal gonadectomy versus retaining gonads after puberty
7	M	Proximal hypospadias, micropenis (at birth)	*CYP17A1* (4 years)	c.1226C>G p.(Pro409Arg) c.1263G>A p.(Ala421=) Comp hetero Sanger	Partial testosterone response to hCG stimulation	17OHD	Staged repair of hypospadias, Inguinal hernia repair (3–4 years)	Adrenal insufficiency	Genetic counseling Parental testing for carrier status and recurrence risk Long-term steroid replacement for adrenal insufficiency Risk of infertility
8	F	Female phenotype (primary amenorrhea at 14 years of age)	*AR* (15 years)	c.2354G>T^b^ p.(Cys785Phe) Hemi Sanger	Normal LH and FSH T ↑	CAIS	Lap bil gonadectomy (16 years)		Genetic counseling Maternal testing for carrier status and recurrence risk Timing of gonadectomy in PAIS and CAIS Prognostic prediction in favor of the female gender in CAIS Risk of gender development incongruent with the sex of rearing in PAIS
9	F	Female phenotype (finding of testis during hernia repair at 2 years of age)	*AR* (4 years)	c.1783delC^b^ p.(Leu595Cysfs*31) Hemi Sanger	Normal LH and FSH T ↑	CAIS	Lap bil gonadectomy (15 years)		
10	F	Female phenotype with clitoromegaly, Bil palpable gonads at groins (virilization at 11 years of age)	*AR* (11 years)	c.2591T>A p.(Leu864Gln) Hemi Sanger	Normal LH and FSH T↑	PAIS	Bil inguinal gonadectomy (12 years)		
11	F	Female phenotype, Bil palpable gonads at groins (antenatal XY fetus and at birth)	*AR* (2 months)	c.2299C>T p.(Pro767Ser) Hemi NGS-targeted gene panel	Normal LH and FSH	CAIS	Lap hernia repair (2 years)		
12	M	Proximal hypospadias, Bifid scrotum (at birth)	*DMRT1* (7 months)	9p24.3p23 deletion arr[GRCh37]9p24.3p23(208454_12427619)x1,9q34.13q34.3(134691007_141018648)x3 Hetero chromosomal microarray	Normal LH, FSH, and T Normal testosterone response to hCG stimulation	PGD	Staged repair of hypospadias, Testicular biopsy (1–2 years)	GDD, ID	Genetic counseling Etiology for syndromic or other somatic presentations Gonadal management of removal vs. preservation with biopsy Risk of gonadal tumors Risk of infertility in males Pregnancy potential in CGD
13	F	Severe atypical genitalia Uni palpable gonad at groin (at birth)	*DMRT1* (6 months)	9p24 deletion arr[GRCh37]9p24.3p23(209,020_12,388,917)x1,9q34.13q34.3(134,715,701_141,005,513) x 3 Hetero chromosomal microarray	Normal LH and FSH, T level incongruent with a female newborn	PGD	Feminizing genitoplasty, Bil gonadectomy (2 years)	ASD, ID	
14	F	Female phenotype (antenatal XY fetus and at birth)	*DMRT1* (10 months)	9p24.3p23 deletion arr[GRCh37]9p24p23(46587 _9425605)x1,9q34.13q34.3(133305614_140755119 × 3 Hetero chromosomal microarray	LH↑FSH↑ Absent testosterone response to hCG stimulation	CGD	Lap bil gonadectomy(2 years)	GDD, ID, ASD Non-paralytic hypotonia	
15	M	Proximal hypospadias, Bifid scrotum (At birth)	*DMRT1* (2 years)	9p24.3p24.1 deletion arr[GRCh37] 9p24.3p24.1 (209020_7222473)x1, 22q13.2q13.33(42552368_ 51178149)x3 Hetero chromosomal microarray	Normal LH,FSH, and T Normal testosterone response to hCG stimulation	PGD	Staged repair of hypospadias (1–2 years) Testicular biopsy (4 years)	ID	
16	F	Female phenotype (Infancy with multiple congenital anomalies)	*DAX1* (18 months)	Xp21 duplication 46,XY, der (22)t(X;22)(p11.4;p11.2) Hemi chromosomal microarray MLPA	LH↑FSH↑	CGD	Lap bil gonadectomy (2 years)	GDD, ID, Lupus nephritis	
17	M	Micropenis, Bil impalpable testes (at birth)	*NR5A1* (3 months)	c.860A>G (VUS) p.(Lys287Arg) Hetero WES	AMH↓↓ T↓↓ Absent testosterone response to hCG stimulation	PGD	Lap bil gonadectomy (1 years)		
18	F	Female phenotype (infancy with multiple congenital anomalies)	*NR5A1* (7 months)	9q33.1q33.3 deletion arr[GRCh37] 9q33.1q33.3(121818843_127626142)x1 Hetero chromosomal microarray	LH↑FSH↑	PGD	Lap bil gonadectomy, Lap hernia repair (2 years)	GDD, ID, ASD, Epilepsy	
19	F	Severe atypical genitalia Uni palpable gonad at groin (at birth)	*NR5A1* (10 years)	c.319C>T p.(Gln107*) Hetero WES	LH↑FSH↑ Partial testosterone response to hCG stimulation	PGD	Lap + inguinal bil gonadectomy (2 years)		
20	M	Proximal hypospadias, Bifid scrotum (at birth)	*WT1* (3 years)	c.1432+4C>T p.? (Splice site change) Hetero Sanger	Normal LH and FSH Normal testosterone response to hCG stimulation	PGD	Staged repair of hypospadias(1–2 years) Testicular biopsy(4 years) Bil orchidectomy(5 years)	Frasier syndrome with MGN	
21	M	Proximal hypospadias, UDT (at birth)	*WT1* (1 years)	c.341C>A^b^ p.(Ser119*) Hetero Sanger	Normal LH and FSH	PGD	Staged repair of hypospadias, Testicular biopsy, Uni orchidectomy (2–3 years)	Bilateral Wilms’ tumors, CKD stage 2	

VUS, variant of uncertain significance; Co-M, Comorbidities; SOR, sex of rearing; M, male; F, female; UDT, undescended testis; Homo, homozygous; Comp hetero, compound heterozygous; Hetero, heterozygous; Hemi, hemizygous; 5α-R2D, 5-alpha reductase type II deficiency; 17βHSD-3, 17-beta hydroxysteroid dehydrogenase III; 17OHD, 17-alpha hydroxylase deficiency; CAIS, complete androgen insensitivity syndrome; PAIS, partial androgen insensitivity syndrome; CGD, complete gonadal dysgenesis; PGD, partial gonadal dysgenesis; TIP, tubularized incised plate; Lap, laparoscopic; Bil, bilateral; Uni, unilateral; GDD, global developmental delay; ASD, atrial septal defect; ID, intellectual disability; MGN, membranous glomerulonephritis; CKD, chronic kidney disease; LH, luteinizing hormone; FSH, follicle stimulating hormone; T, testosterone; hCG, human chorionic gonadotrophin; AMH, antimullerian hormone; USP, urine steroid profile; NGS, next-generation sequencing.

^a^ Variant either pathogenic or likely pathogenic unless specified as VUS.

^b^ Mutations not reported in gnomAD or HGMD.

Four patients had androgen receptor (*AR*) gene variants, resulting in CAIS (*n* = 3) and PAIS (*n* = 1). All three CAIS patients had female-typical phenotypes with palpable testes (*n* = 1) and impalpable intraabdominal testes (*n* = 2). The PAIS patient presented with a deepening of voice and clitoromegaly at the onset of puberty.

Of the seven patients who had genetic variants impairing androgen synthesis, five had *SRD5A2* variants resulting in 5-alpha reductase type II deficiency (5α-R2D). Two patients had variants in *HSD17B3* and *CYP17A1* genes, resulting in 17-beta hydroxysteroid dehydrogenase III (17βHSD-3) deficiency and 17-alpha hydroxylase deficiency (17OHD), respectively. All but one in this subgroup presented with proximal hypospadias and micropenis at birth. The exception was the patient with 17βHSD-3 deficiency, who was born with a predominant female phenotype with bilateral palpable testes in the enlarged labia.

Ten patients had GD. Two had CGD with female-typical phenotypes and bilateral intraabdominal streak gonads. The remaining eight patients had PGD presenting with proximal hypospadias (*n* = 5), predominant female phenotype with bilateral intraabdominal dysgenetic gonads (*n* = 1), and severe atypical external genitalia (*n* = 2). Their genetic diagnoses were *NR5A1* gene variants (*n* = 3), *DMRT1* gene deletion (*n* = 4), *WT1* gene variants (*n* = 2), and *DAX1* (*NR0B1*) gene duplication (*n* = 1). Eight out of 10 patients had comorbidities involving other systems. Six had varying severities of intellectual disability or developmental delay. One had the syndromic DSD Frasier syndrome.

Of the whole group, 20 patients underwent one or more surgical procedures, including hypospadias repair (*n* = 10), gonadal removal (*n* = 11), gonadal biopsy (*n* = 4), hernia repair (*n* = 4), orchidopexy (*n* = 1), and feminizing genitoplasty (*n* = 1). A total of five patients had neoplasms in one or both gonads (Table 2). Our gonadal management was based on a risk-stratification strategy. No biopsy or removal was performed in male patients with disorders of androgen synthesis. We preserved gonads in CAIS until after puberty. Removal of gonads in PAIS was considered when female gender development was evident. For GD, we removed the non-functioning streak gonads or dysgenetic gonads in female patients. We preserved the dysgenetic but functioning testes in male patients with prepubertal and postpubertal biopsy surveillance.

**Table 2:** Summary of five patients with gonadal germ cell neoplasms.

Patient No.	Age at the time of gonadectomy	Diagnosis	Gonadal features and locations	Histology
8	16 years	CAIS with *AR* variant	Bilateral intraabdominal testes	GCNIS in bilateral testes
14	30 months	CGD with *DMRT1* deletion	Bilateral intraabdominal streak gonads	GB in the right gonad
16	31 months	CGD with *DAX1 (NR0B1)* duplication	Bilateral intraabdominal streak gonads	GB in bilateral gonads
19	20 months	PGD with *NR5A1* variant	Right intraabdominal dysgenetic gonad, left inguinal dysgenetic gonad	Immature teratoma + GCNIS in the left gonad
20	4 years	PGD with *WT1* variant, Frasier syndrome	Bilateral fully descended dysgenetic testes in the scrotum	GCNIS in bilateral testes

CAIS, complete androgen insensitivity syndrome; CGD, complete gonadal dysgenesis; PGD, partial gonadal dysgenesis; GB, gonadoblastoma; GCNIS, germ cell neoplasia *in situ*.

Seven patients were found to inherit the genetic alterations from their parents. None of the patients was born to consanguineous parents. The seven patient cases involved *SRD5A2* variants (*n* = 4), *DMRT1* deletion (*n* = 1), *CYP17A1* variant (*n* = 1), and *AR* variant (*n* = 1).

## Discussion

The present study reported a cohort of children with 46,XY DSD, with genetic diagnoses accounting for their DSD and the surgical treatment received by them. The causes of DSD are, in general, genetically determined. Being highly heterogeneous in etiology, the genetic diagnostic rates reported in 46,XY DSD by studies using single-gene sequencing were relatively low (3, 10). In recent years, NGS has rapidly expanded its applications worldwide. NGS allows massively parallel sequencing to simultaneously assess a targeted DSD-related gene panel, or the entire protein-coding region as in WES (1, 7, 8). Recent studies incorporating NGS techniques in their diagnostic strategy have reported genetic diagnostic rates of 31%–64% in children with 46,XY DSD (11–13). WES is particularly useful in detecting novel or very rare genetic causes for 46,XY DSD (14, 15). In a large North African cohort study, the authors employed WES and were able to identify 30 pathogenic/likely pathogenic variants involving 25 different genes, which had not been reported previously as the etiological causes for 46,XY DSD (15). Molecular genetic tests play an increasingly important role in DSD management. A genetic diagnosis that can be reached in up to 50% of 46,XY DSD cases allows for prognostic predictions, genetic counseling, and individualized management (16).

The genetic diagnosis of 5α-R2D in five of our patents who presented with proximal hypospadias and micropenis supported the decision of the male sex of rearing, given their high likelihood of developing male gender identity (1, 16, 17). The diagnosis of 5α-R2D had prompted the use of the topical dihydrotestosterone (DHT) cream before hypospadias surgery. It is beyond the scope of this study to investigate the effects of the DHT cream in boys with 46,XY DSD before hypospadias surgery. There are reports in the literature that have supported its use in patients with 5α-R2D (16, 18–20). Our surgical approach to correct hypospadias for children with DSD was similar to how we managed isolated hypospadias without DSD issues in terms of surgical techniques and timing of surgery. Most of our patients with proximal hypospadias in the present study had severe ventral curvature, requiring the transection of the urethral plate, and underwent staged repair. Staged repair is the preferred approach in the masculinizing procedure for correcting severe hypospadias and ventral curvature in DSD patients reared as boys (16, 18, 19).

The identification of a *CYP17A1* variant causing 17OHD in another patient with proximal hypospadias had compelled additional investigations for adrenal function (21), and adrenal insufficiency was subsequently diagnosed. The parents of this patient and those of the other four patients with 5α-R2D were found to be carriers of the variant genes. As both 17OHD and 5α-R2D are inherited in an autosomal recessive pattern, the rate of recurrence risk is 25% for the next 46,XY offspring of these families. Despite the masculinizing procedure to improve the cosmetic and functional aspects of the external genitalia, patients with 17OHD and 5α-R2D have a very high risk of sub- or infertility due to oligospermia, azoospermia, and increased viscosity of semen (16, 22). Early development of a long-term management plan to preserve spermatogenesis, combined with the use of assisted reproductive technology in adulthood, has been recommended to enhance the fertility potential of such patients (22).

Ten patients in our cohort had either PGD or CGD. The genetic alterations found in this study involved *NR5A1, DMRT1, WT1*, and *DAX1,* which all have been known to be associated with GD (6, 8, 23). Among the DSD subgroups or specific diagnoses, 46,XY GD is associated with the highest risk of gonadal germ cell neoplasm, with the reported risk rates varying between 8% and 54% (23–25). In a recent multi-institutional study on 1,040 DSD patients in Europe, the overall rate of risk of gonadal germ cell neoplasm in 46,XY DSD was reported to be 14% (24). The highest risk rate of 36% was found in patients with 46,XY GD, with gonadoblastoma (GB) or germ cell neoplasia *in situ* (GCNIS) being the most common type of gonadal neoplasia detected (24). Our findings were in agreement with the literature, and we found four (40%) of our patients with GD had gonadal germ cell neoplasms of either GB or GCNIS diagnosed at their age of 20 months to 4 years. Notably, the only two patients with CGD in our cohort were both found to have GB in their intraabdominal streak gonads. GB and GCNIS are considered to be premalignant *in situ* neoplasms, which would progress to frank malignant lesions if left untreated (23). The molecular diagnosis of GD had prompted the consideration of risk-stratified gonadal management in this subgroup of patients. Among the five patients with GD who were reared as girls in this study, we removed their streak or dysgenetic gonads. Such gonads were either non-functioning or discordant in the production of the sex hormone. We preserved the dysgenetic but functioning testes in patients reared as boys with pre- and postpubertal biopsy surveillance in an approach similar to our management for patients with 45,X/46,XY (26).

Three of our patients with GD had *NR5A1* variants. The *NR5A1* gene, located on the long arm of chromosome 9, encodes a transcriptional factor called steroidogenic factor 1, which controls the activity of several genes related to sex determination and the adrenal gland (27). A recent study analyzing global molecular diagnostic cohorts reported that *AR, SRD5A2*, and *NR5A1*, which were the most common genes detected with mutations, was the etiological cause for 46,XY DSD, and the highest rate of frequency of mutation in *NR5A1* reported in cohort studies was 22% (28). Affected individuals present with a broad spectrum of phenotypes ranging from male-typical phenotype with isolated infertility, hypospadias, and severe atypical genitalia to female-typical phenotype (27). Our three patients with *NR5A1* variants had different phenotypes. One had a female phenotype. Another patient had severe atypical genitalia with GCNIS and immature teratoma in one of the dysgenetic gonads. No feminizing procedure was performed for this female-reared PGD patient. Her parents accepted the persistent ambiguity of external genitalia to preserve all anatomical options available when the patient's gender identity could be ascertained. Of the three *NR5A1* patients in our cohort, one was found to have gonadal germ cell neoplasm. In contrast, a study on the so far largest international cohort of individuals with *NR5A1* variants found only two patients (2/128, 1.6%) with gonadal germ cell tumors at the ages of 3 and 21 years, respectively (29). The authors, however, exercised caution to draw any premature conclusion as many study individuals had underwent gonadectomy at an early age without uniform histological examinations of the gonads, and many others were still young. The authors suggested more long-term studies to assess tumor risk associated with *NR5A1* variants in 46,XY DSD (29).

The only case of VUS reported in the present study was associated with *NR5A1,* and the patient was born with micropenis and bilateral impalpable testes. Based on the ACMG guidelines, the variant was classified as VUS as the mutation was not located in the critical domain nor in the mutational hotspot, and had not been described in the literature previously. The antimullerian hormone level was very low and testosterone response to the prolonged human chorionic gonadotrophin (hCG) stimulation test was negligible. Laparoscopy revealed bilateral tiny gonads at the location of deep rings, following which bilateral gonadectomy was performed. Histology showed fibrovascular tissue, with structures resembling fallopian tubes, and the overall findings were compatible with GD. The phenomenon of gonadal regression is considered part of the spectrum of GD (30, 31).

In our cohort, a case of *DAX1* duplication and four cases of *DMRT1* deletion were found to be the genetic etiology for 46,XY GD. In contrast, recent genetic studies in over 250 children with 46,XY DSD did not identify a single case of *DMRT1* gene deletion (11–13). The *DMRT1* gene is located at the short arm of chromosome 9, and it encodes a transcription factor that regulates testicular differentiation. Apart from a wide spectrum of DSD phenotypes, 46,XY individuals with *DMRT1* deletion may also present with somatic features related to the 9p deletion syndrome, such as mental retardation, craniofacial dysmorphism, and delayed motor development (32, 33). *DAX1*, also known as the *NR0B1* gene, is located at Xp21. Duplication of the *DAX1* gene inhibits the expression of other sex-determining genes such as *SOX9* and *SRY*, resulting in a failure of fetal testis development. The clinical manifestations of *DMRT1* deletion and *DAX1* duplication are influenced by the underlying mechanism, which can be purely a terminal chromosomal deletion/duplication, or a more complex chromosomal rearrangement such as unbalanced translocation. The patient's somatic phenotype also depends on the size of chromosomal imbalance and critical genes being involved. Similar to 9p deletion, Xp21 duplication is associated with mental retardation, neurodevelopmental delay, muscular dystrophy, and craniofacial dysmorphism (34–36).

The five patients in our study affected by *DMRT1* deletion or *DAX1* duplication had varying severity of intellectual disability, requiring attendance at special schools. The patient with *DAX1* duplication had a global developmental delay, intellectual disability, atrial septal defect, and lupus nephritis. Among the four patients with *DMRT1* deletion, two had evidence of brain atrophy shown in MRI and experienced motor function development delay and hypotonia, requiring physiotherapy and occupational therapy training. Two patients also had associated cardiac structural defects. Testicular biopsies were performed in addition to hypospadias surgery for the two patients reared as boys. In both patients, histology showed remarkable findings of seminiferous tubules composed of only Sertoli cells, without germ cells noted. Such findings suggested a high risk of infertility. Notably, the two patients with CGD affected by *DMRT1* deletion and *DAX1* duplication, respectively, had uterus, and theoretically had pregnancy potential via the ovum donation. The only female patient in the present study who underwent feminizing genitoplasty had PGD due to *DMRT1* deletion. The patient underwent reduction clitoroplasty, which was irreversible in nature. Multiple counseling sessions were conducted with parents by an MDT to reach a shared decision.

The *WT1* gene encodes a transcription factor that plays a key role in kidney development and gonadal differentiation. Mutations in the *WT1* gene are associated with life-threatening nephropathy, GD, and Wilms’ tumor (37). One of our patients with *WT1* gene mutation had bilateral Wilms’ tumors. *WT1* gene mutation is also known to be the etiology for syndromic DSD such as Denys–Drash syndrome and Frasier syndrome (23, 37). A molecular diagnosis of Frasier syndrome in one of our patients with proximal hypospadias had prompted the need for testicular biopsies, which demonstrated germ cell neoplasia in situ. Subsequently, bilateral orchidectomy was performed, and histology confirmed a bilateral involvement of germ cell neoplasms.

Of the four patients with *AR* variants, three had CAIS and one had PAIS. The PAIS patient had been reared as female without a significant past medical history until she developed a deepening of voice and clitoromegaly at the onset of puberty. Full disclosure of the underlying DSD condition was given to the patient. Gonadotropin-releasing hormone (GnRH) analog was prescribed to control virilization and allow enough time for the patient and family to make a decision. The patient was mentally mature and consistent in identifying as female. She repeatedly expressed her wish to have the testes removed to prevent further progression of virilization. Bilateral gonadectomy was performed at the age of 12 years with the patient's informed consent. Clitoromegaly showed a reduction after gonadectomy, although her deepened voice did not show improvement. We did not offer clitoral reduction surgery for this patient as we did not see any benefits of considering such a surgery in an adolescent patient, and therefore, we left the decision to the patient in her adulthood. While patients with CAIS always identify themselves as female, individuals with PAIS may develop a gender identity opposite to the sex assigned to them in infancy (2).

The latest patient with CAIS during our study period presented prenatally with discordant findings of XY fetus by amniocentesis and female phenotype by morphology scan. The molecular diagnosis supported unequivocally the female sex of rearing. Following contemporary recommendations, we preserved the testes of this patient for the benefits of endogenous conversion of testosterone to estrogen via aromatization during puberty and advised postpubertal gonadectomy (16, 19, 38). The patient subsequently developed inguinal hernias. A laparoscopic extraperitoneal closure of the bilateral deep rings was performed using our standard techniques (39), with the two testes being placed in the inguinal canals to facilitate subsequent physical examination and ultrasound surveillance.

One of our patients with CAIS had GCNIS in the bilateral testes by 16 years of age. A recent systematic review on the risk of gonadal tumors in CAIS reported occurrence rates of 6% and 1.3% for premalignant and malignant lesions, respectively (40). The authors found that 82% of the premalignant lesions were reported in patients older than 12 years and all malignant lesions occurred in patients older than 20 years (40). Although the overall tumor risk remains low, it is important to ensure a full understanding of the risks and benefits of preserving the testes among adolescent patients with CAIS.

The only patient in our cohort who had not undergone any surgery was the one with 17βHSD-3 deficiency. Although this patient was born with a predominant female phenotype, virilization at the onset of puberty was anticipated (41). The patient’s parents received full disclosure of the underlying diagnosis. The pros and cons of performing prepubertal gonadectomy vs. retaining the gonads until the patient's gender identity becomes clear were discussed. The patient was still a toddler. The MDT and the patient’s parents had yet to arrive at any decision on surgical intervention. Arrangements had been made for parental testing for determining carrier status, but the results were not available at the time of data collection. This case was the only one in our cohort in which the parents were non-Chinese and consanguineous.

Our study was limited by its retrospective nature over a long study period in which the application of genetic testing had evolved significantly. Given the lack of a standardized protocol, our cohort probably missed some patients who would have been included had modern-day genetic tests been performed. The reported proportions of the three diagnostic categories did not reflect the true proportions of the 46,XY DSD patients we had encountered in clinical practice as only those with molecular diagnoses were included. Parental testing could have been significantly influenced by personal factors and the availability of such services at the time.

The present study reported the genetic diagnoses and surgical management of a heterogeneous cohort of children with 46,XY DSD. Molecular genetic diagnosis enhances the understanding of etiology and improves diagnostic accuracy. With modern technology, a genetic diagnosis can be reached expectedly in up to 50% of 46,XY DSD cases, and the diagnostic rate may continue to rise. Surgeons involved in MDT care for DSD should be aware of the increasing role of genetic testing. The role of the surgeon in DSD care is not limited to operative skills. A molecular diagnosis can enhance the input of the surgeon in advising on the sex of rearing, formulating a condition-specific individualized surgical plan on gonadal management and genital surgery, partnering with other disciplines in counseling patients and their parents throughout the journey.

## Data Availability

The datasets presented in this article are not readily available because data are not to be shared outside our institution according to our prevailing policy. Requests to access the datasets should be directed to the corresponding author.

## References

[1] Achermann JC Domenice S Bachega TA Nishi MY Mendonca BB. Disorders of sex development: effect of molecular diagnostics. Nat Rev Endocrinol. (2015) 11(8):478–88. 10.1038/nrendo.2015.69 25942653

[2] Lee PA Houk CP Ahmed SF Hughes IA, International Consensus Conference on Intersex Organized by the Lawson Wilkins Pediatric Endocrine Society and the European Society for Paediatric Endocrinology. Consensus statement on management of intersex disorders. Pediatrics. (2006) 118(2):e488–500. 10.1542/peds.2006-0738 16882788

[3] Lek N Miles H Bunch T Pilfold-Wilkie V Tadokoro-Cuccaro R Davies J Low frequency of androgen receptor gene mutations in 46 XY DSD, and fetal growth restriction. Arch Dis Child. (2014) 99(4):358–61. 10.1136/archdischild-2013-305338 24366239

[4] Nixon R Cerqueira V Kyriakou A Lucas-Herald A McNeilly J McMillan M Prevalence of endocrine and genetic abnormalities in boys evaluated systematically for a disorder of sex development. Hum Reprod. (2017) 32(10):2130–7. 10.1093/humrep/dex280 28938747 PMC5850224

[5] Baetens D Mladenov W Delle Chiaie B Menten B Desloovere A Iotova V Extensive clinical, hormonal and genetic screening in a large consecutive series of 46,XY neonates and infants with atypical sexual development. Orphanet J Rare Dis. (2014) 9:209. 10.1186/s13023-014-0209-2 25497574 PMC4271496

[6] Eggers S Sadedin S van den Bergen JA Robevska G Ohnesorg T Hewitt J Disorders of sex development: insights from targeted gene sequencing of a large international patient cohort. Genome Biol. (2016) 17(1):243. 10.1186/s13059-016-1105-y 27899157 PMC5126855

[7] Byers HM Fossum M Wu HY. How geneticists think about differences/disorders of sexual development (DSD): a conversation. J Pediatr Urol. (2020) 16(6):760–7. 10.1016/j.jpurol.2020.08.015 32893165

[8] Audi L Ahmed SF Krone N Cools M McElreavey K Holterhus PM Genetics in endocrinology: approaches to molecular genetic diagnosis in the management of differences/disorders of sex development (DSD): position paper of EU COST action BM 1303 ‘DSDnet’. Eur J Endocrinol. (2018) 179(4):R197–206. 10.1530/EJE-18-0256 30299888 PMC6182188

[9] Richards S Aziz N Bale S Bick D Das S Gastier-Foster J Standards and guidelines for the interpretation of sequence variants: a joint consensus recommendation of the American College of Medical Genetics and Genomics and the Association for Molecular Pathology. Genet Med. (2015) 17(5):405–24. 10.1038/gim.2015.30 25741868 PMC4544753

[10] Akcay T Fernandez-Cancio M Turan S Güran T Audi L Bereket A. AR and SRD5A2 gene mutations in a series of 51 Turkish 46,XY DSD children with a clinical diagnosis of androgen insensitivity. Andrology. (2014) 2(4):572–8. 10.1111/j.2047-2927.2014.00215.x 24737579

[11] Ata A Özen S Onay H Uzun S Gökşen D Özkınay F A large cohort of disorders of sex development and their genetic characteristics: 6 novel mutations in known genes. Eur J Med Genet. (2021) 64(3):104154. 10.1016/j.ejmg.2021.104154 33516834

[12] Xu Y Wang Y Li N Yao R Li G Li J New insights from unbiased panel and whole-exome sequencing in a large Chinese cohort with disorders of sex development. Eur J Endocrinol. (2019) 181(3):311–23. 10.1530/EJE-19-0111 31277073

[13] Jacobson JD Willig LK Gatti J Strickland J Egan A Saunders C High molecular diagnosis rate in undermasculinized males with differences in sex development using a stepwise approach. Endocrinology. (2020) 161(5):bqz015. 10.1210/endocr/bqz015 32010941

[14] Rjiba K Mougou-Zerelli S Hamida IH Saad G Khadija B Jelloul A Additional evidence for the role of chromosomal imbalances and SOX8, ZNRF3 and HHAT gene variants in early human testis development. Reprod Biol Endocrinol. (2023) 21(1):2. 10.1186/s12958-022-01045-7 36631813 PMC9990451

[15] Zidoune H Ladjouze A Chellat-Rezgoune D Boukri A Dib SA Nouri N Novel genomic variants, atypical phenotypes and evidence of a digenic/oligogenic contribution to disorders/differences of sex development in a large north African cohort. Front Genet. (2022) 13:900574. 10.3389/fgene.2022.900574 36110220 PMC9468775

[16] Wisniewski AB Batista RL Costa EMF Finlayson C Sircili MHP Dénes FT Management of 46,XY differences/disorders of sex development (DSD) throughout life. Endocr Rev. (2019) 40(6):1547–72. 10.1210/er.2019-00049 31365064

[17] Costa EM Domenice S Sircili MH Inacio M Mendonca BB. DSD due to 5α-reductase 2 deficiency—from diagnosis to long term outcome. Semin Reprod Med. (2012) 30(5):427–31. 10.1055/s-0032-1324727 23044880

[18] Ochi T Ishiyama A Yazaki Y Murakami H Takeda M Seo S Surgical management of hypospadias in cases with concomitant disorders of sex development. Pediatr Surg Int. (2019) 35(5):611–7. 10.1007/s00383-019-04457-6 30762107

[19] Hemesath TP de Paula LCP Carvalho CG Leite JCL Guaragna-Filho G Costa EC. Controversies on timing of sex assignment and surgery in individuals with disorders of sex development: a perspective. Front Pediatr. (2019) 6:419. 10.3389/fped.2018.00419 30687685 PMC6335325

[20] Odame I Donaldson MD Wallace AM Cochran W Smith PJ. Early diagnosis and management of 5 alpha-reductase deficiency. Arch Dis Child. (1992) 67(6):720–3. 10.1136/adc.67.6.720 1626992 PMC1793798

[21] Auchus RJ. Steroid 17-hydroxylase and 17,20-lyase deficiencies, genetic and pharmacologic. J Steroid Biochem Mol Biol. (2017) 165(Pt A):71–8. 10.1016/j.jsbmb.2016.02.002 26862015 PMC4976049

[22] Markouli M Michala L. Fertility potential in 5α-reductase type 2 deficient males. J Pediatr Urol. (2023) 19(1):108–14. 10.1016/j.jpurol.2022.09.002 36153242

[23] McCann-Crosby B Mansouri R Dietrich JE McCullough LB Sutton VR Austin EG State of the art review in gonadal dysgenesis: challenges in diagnosis and management. Int J Pediatr Endocrinol. (2014) 2014(1):4. 10.1186/1687-9856-2014-4 24731683 PMC3995514

[24] Slowikowska-Hilczer J Szarras-Czapnik M Duranteau L Rapp M Walczak-Jedrzejowska R Marchlewska K Risk of gonadal neoplasia in patients with disorders/differences of sex development. Cancer Epidemiol. (2020) 69:101800. 10.1016/j.canep.2020.101800 32905884

[25] Wolffenbuttel KP Hersmus R Stoop H Biermann K Hoebeke P Cools M Gonadal dysgenesis in disorders of sex development: diagnosis and surgical management. J Pediatr Urol. (2016) 12(6):411–6. 10.1016/j.jpurol.2016.08.015 27769830

[26] Wong YS Pang KKY Tam YH. Surgery in Chinese children affected by 45,X/46,XY disorders of sex development: a 20-year experience in a single center. J Pediatr Surg. (2022) 57(7):1398–403. 10.1016/j.jpedsurg.2021.05.018 34167801

[27] Fabbri-Scallet H de Sousa LM Maciel-Guerra AT Guerra-Júnior G de Mello MP. Mutation update for the NR5A1 gene involved in DSD and infertility. Hum Mutat. (2020) 41(1):58–68. 10.1002/humu.23916 31513305

[28] Jiali C Huifang P Yuqing J Xiantao Z Hongwei J. Worldwide cohort study of 46, XY differences/disorders of sex development genetic diagnoses: geographic and ethnic differences in variants. Front Genet. (2024) 15:1387598. 10.3389/fgene.2024.1387598 38915825 PMC11194351

[29] Kouri C Sommer G de Lapiscina IM Elzenaty RN Tack LJW Cools M Clinical and genetic characteristics of a large international cohort of individuals with rare NR5A1/SF-1 variants of sex development. EBioMedicine. (2024) 99:104941. 10.1016/j.ebiom.2023.104941 38168586 PMC10797150

[30] Cools M Pleskacova J Stoop H Hoebeke P Van Laecke E Drop SL Gonadal pathology and tumor risk in relation to clinical characteristics in patients with 45,X/46,XY mosaicism. J Clin Endocrinol Metab. (2011) 96(7):E1171–80. 10.1210/jc.2011-0232 21508138

[31] Andrade JGR Fabbri-Scallet H Dos Santos AP Cools M Werner R Hiort O Clinical findings and follow-up of 46,XY and 45,X/46,XY testicular dysgenesis. Sex Dev. (2019) 13(4):171–7. 10.1159/000504239 31816618

[32] Barbaro M Balsamo A Anderlid BM Myhre AG Gennari M Nicoletti A Characterization of deletions at 9p affecting the candidate regions for sex reversal and deletion 9p syndrome by MLPA. Eur J Hum Genet. (2009) 17(11):1439–47. 10.1038/ejhg.2009.70 19417767 PMC2986678

[33] Lopes AM Aston KI Thompson E Carvalho F Gonçalves J Huang N Human spermatogenic failure purges deleterious mutation load from the autosomes and both sex chromosomes, including the gene DMRT1. PLoS Genet. (2013) 9(3):e1003349. 10.1371/journal.pgen.1003349 23555275 PMC3605256

[34] Mehawej C Maalouf JE Abdelkhalik M Mahfouz P Chouery E Megarbane A. CNV analysis through exome sequencing reveals a large duplication involved in sex reversal, neurodevelopmental delay, epilepsy and optic atrophy. Genes (Basel). (2024) 15(7):901. 10.3390/genes15070901 39062680 PMC11275410

[35] Sukumaran A Desmangles JC Gartner LA Buchlis J. Duplication of dosage sensitive sex reversal area in a 46, XY patient with normal sex determining region of Y causing complete sex reversal. J Pediatr Endocrinol Metab. (2013) 26(7–8):775–9. 10.1515/jpem-2012-0354 23612644

[36] Zheng XQ Zhou QL Gu W. 46,XY disorders of sex development and muscular dystrophy caused by Xp21 duplication: a case report and literature review. Transl Pediatr. (2024) 13(11):2088–96. 10.21037/tp-24-327 39649651 PMC11621888

[37] Arroyo-Parejo Drayer P Seeherunvong W Katsoufis CP DeFreitas MJ Seeherunvong T Chandar J Spectrum of clinical manifestations in children with WT1 mutation: case series and literature review. Front Pediatr. (2022) 10:847295. 10.3389/fped.2022.847295 35498778 PMC9051246

[38] Mouriquand PD Gorduza DB Gay CL Meyer-Bahlburg HF Baker L Baskin LS Surgery in disorders of sex development (DSD) with a gender issue: if (why), when, and how? J Pediatr Urol. (2016) 12(3):139–49. 10.1016/j.jpurol.2016.04.001 27132944

[39] Tam YH Lee KH Sihoe JD Chan KW Wong PY Cheung ST Laparoscopic hernia repair in children by the hook method: a single-center series of 433 consecutive patients. J Pediatr Surg. (2009) 44(8):1502–5. 10.1016/j.jpedsurg.2008.10.071 19635295

[40] Barros BA Oliveira LR Surur CRC Barros-Filho AA Maciel-Guerra AT Guerra-Junior G. Complete androgen insensitivity syndrome and risk of gonadal malignancy: systematic review. Ann Pediatr Endocrinol Metab. (2021) 26(1):19–23. 10.6065/apem.2040170.085 33819955 PMC8026333

[41] George MM New MI Ten S Sultan C Bhangoo A. The clinical and molecular heterogeneity of 17βHSD-3 enzyme deficiency. Horm Res Paediatr. (2010) 74(4):229–40. 10.1159/000318004 20689261

